# A Retrospective Analysis of Acute Gastroenteritis Agents in Children Admitted to a University Hospital Pediatric Emergency Unit

**DOI:** 10.5812/jjm.9148

**Published:** 2014-04-01

**Authors:** Suat Bicer, Defne Col, Gulay Ciler Erdag, Tuba Giray, Yesim Gurol, Gulden Yilmaz, Ayca Vitrinel, Berna Ozelgun

**Affiliations:** 1 Department of Child Health and Pediatrics, Faculty of Medicine, Yeditepe University, Istanbul, Turkey; 2 Department of Medical Microbiology, Faculty of Medicine, Yeditepe University, Istanbul, Turkey

**Keywords:** Acute gastroenteritis, Child, Immunochromatography, Norovirus, Rotavirus

## Abstract

**Background:**

Acute gastroenteritis is responsible observed in all age groups, especially infants and children. The etiology and clinical course of acute gastroenteritis may vary with age and etiological agents. In developing countries, the morbidity and mortality associated with infectious diarrhea is higher in children younger than five-years-of-age.

**Objectives:**

The aim of this study was to determine the prevalence and seasonal distribution of the major agents of acute gastroenteritis in children who were admitted to a Turkish university hospital pediatric emergency unit during 2009.

**Patients and Methods:**

Seasonal distribution within a one year period and quantitative distribution were analyzed with demographic data and laboratory findings. A total of 644 subjects were enrolled in the study, with a mean age of 4.14 years. Pathogens were detected in 183 (28.4%) stool samples in children less than 16 years, admitted with acute gastroenteritis.

**Results:**

Pathogens were detected in 184 (28.4%) stool samples. The age distributions of the cases were 0 - 24 months (n = 59), 2 - 5 years (n = 100), and > 5 years (n = 25). The detection rate of rotavirus, norovirus and adenovirus were; 12.7% (75/588), 9.8% (51/520) and 4.9% (28/575), respectively. Bacterial agents were detected in 36 cases. The main agent was norovirus in the 0 - 24 months group (n = 25, 42.4%), and rotavirus for ages 2 - 5 years (n = 43, 43%) and > 5 years. On the monthly distribution, cases of rotavirus were found to be more frequent in the first four months of the year.

**Discussion:**

Viruses were the major pathogens in all age groups. Norovirus was the leading pathogen in the first two years. For the age groups 2 - 5 years and 6 - 16 years, rotavirus was the major pathogen.

## 1. Background

Acute gastroenteritis is observed in all age groups, but the etiology and clinical course of the disease may vary with age and etiological agents. In developing countries, the morbidity and mortality associated with infectious diarrhea is higher in children younger than five-years-of-age (1). Acute gastroenteritis is caused by viruses, bacteria, and protozoa (1), of these, rotavirus, norovirus, and enteric adenovirus are the major agents of viral gastroenteritis, particularly in children. Rotavirus is the leading cause of acute gastroenteritis in infants and young children (2, 3). Viral gastroenteritis occurs in winter worldwide, with transmission mainly through the fecal-oral route (1-3). The present study monitored the incidence and etiology of acute viral gastroenteritis, and its relationship with patients’ age and season. This epidemiological survey was performed over a 12-month period (January 2009 to January 2010) at a university hospital pediatric emergency clinic in Istanbul.

## 2. Objectives

The aim of this study was to determine the prevalence and seasonal distribution of the major agents of acute gastroenteritis in children who were admitted to a university hospital pediatric emergency unit during 2009.

## 3. Patients and Methods

The patients with acute gastroenteritis who were admitted to the Pediatric Emergency Unit of Yeditepe University Hospital, Istanbul, Turkey, were evaluated retrospectively. The study protocol was approved by the Local Ethical Committee of Yeditepe University Hospital.

### 3.1. Case Definition With Inclusion and Exclusion Criteria

Acute diarrhea was defined as loose stools, at least three times in a 24-hour period, or an episode of forceful vomiting and loose stools, lasting less than 14 days (2). Exclusion criteria included; diarrhea lasting more than two weeks, history of recurrent diarrhea, history of noninfectious gastrointestinal disease (e.g. inflammatory bowel disease, celiac disease), concomitant diseases (eg bronchiolitis, acute upper and lower respiratory tract infections, or urinary tract infection), or any other underlying disorders. Demographic and laboratory data were extracted from the hospital database. The files of all patients, who were hospitalized due to acute diarrhea, were reviewed retrospectively.

### 3.2. Sample Preparation and Laboratory Methods

Fresh stool samples were used to detect the agents. The samples where collection exceeded one hour were not included in the study. The samples were delivered to the microbiology laboratory of the Yeditepe University Hospital with Carry-Blair transport broth, (Salubris Turkey, Istanbul) within ten minutes. Microscopic examination of the stools and viral antigen tests were carried out within an hour. Adenovirus serotypes 40 and 41 and rotavirus were studied with a CerTest Rota-Adeno Blister Test (CerTest, Biotec, Spain), which is a qualitative immunochromatographic assay method. Norovirus was studied with an immunochromatographic assay method (RidaQuick NoV, r-biopharm, Germany).

 In this test, the membrane in the test band was first coated with mouse monoclonal antibodies against viral antigens. During the test, a pre-colored conjugate that had previously been dried was reacted with the sample. Afterwards, the mixture moved forward on the membrane by means of capillary action. The colored particles were replaced as the sample moved along the test membrane. If the result was positive, the specific antibodies on the membrane captured the colored particles.

Samples with leukocytes found in the stools, fever, negative viral antigen tests, or diarrhea with bloody-mucus, were cultured for the presence of; *Salmonella* spp., *Shigella* spp. and *Campylobacter jejuni*. The culture samples that were supplied to the Yeditepe University, Faculty of Medicine, microbiology laboratory were examined when convenient by a microbiology resident. Cases of possible large intestine/colonic diarrhea with complaints of bloody-mucoid diarrhea, tenesmus, abdominal pain, and fever, were studied for the presence of *Entamoeba histolytica* antigens using an ELISA method.

### 3.3. Findings and Statistical Analysis

Seasonal divergence within a year and quantitative distribution were analyzed with demographic data and laboratory findings. Agents were set for the different age groups: 2 years, 2 - 5 years, and older than 5 years. The stool samples were not investigated for less common viral agents (such as astrovirus, calicivirus) or *Cryptosporidium parvum*. Pathogens of acute gastroenteritis were evaluated in 644 children between 3 months to 16 years. Rotavirus, adenovirus and norovirus antigen tests were performed for 588, 575 and 520 cases, respectively. *E. histolytica* antigen test and stool culture were performed for 144 and 82 cases, respectively.

The statistical computations were carried out using SPSS (version 18) statistical package (SPSS Inc., Chicago). The results are given as mean values and standard deviations (SD) or percentages. For comparison of seasonal and age distributions among the cases, we categorized the subjects into different subgroups according to the etiological agents. The statistical findings were analyzed with an ANOVA test to compare the quantitative variables. For comparison of percentages as well as specific pair-wise comparisons, a χ2 test was used. The statistical significance was defined as P < 0.05 for two-tailed analysis in comparing overall groups and the least significant difference procedure.

## 4. Results

A total of 644 subjects were enrolled in the study, with a mean age of 4.14 years. There were no significant differences for mean ages between the case groups according to etiological agents (P > 0.05, ANOVA). Pathogens were detected in 183 (28.4%) stool samples from 644 children less than 16 years admitted with acute gastroenteritis. Among the samples, 461 (71.6%) were negative for all tested pathogens. Viral and bacterial agents were detected in 154 and 36 cases, respectively. Rotavirus was the most frequent pathogen, with a rate of 12.7% (n = 75/588). Norovirus was the second etiological agent accounting for 51 samples with a rate of 9.8% (n = 51/520). Another enteric pathogen was adenovirus with a rate of 4.7% (n = 28/594). Bacterial enteric pathogens of acute gastroenteritis were *Salmonella* spp. and *C. jejuni*, with detection rates of 25.6% (n = 21/82) and 18.3% (n = 15/82), respectively. 

The rate of *E. histolytica* was 2.8% (n = 4/144). *Shigella* spp. was not detected in any of the stool samples (Table 1). Coinfections were present in 11 cases (1.9%). There was virus-virus coinfection in 10 cases. The most frequent coinfection was rotavirus-adenovirus (6 cases). In three cases norovirus and adenovirus were present, and in one case rotavirus and norovirus were found together as a coinfection. There were two cases of coinfection with bacteria-virus: in one case - rotavirus, adenovirus and *Salmonella* spp., and in the other - adenovirus and *Salmonella* spp. (Table 2). 

**Table 1.** Etiology of Acute Gastroenteritis in 594 Children^a^

Pathogen	Samples, No.	Total Samples, No. (%)
**Rotavirus ** ^**b**^	588	75 (12.7)
**Norovirus ** ^**b**^	520	51 (9.8)
**Adenovirus ** ^**b**^	575	28 (4.9)
***Salmonella*** ** spp.**	82	21 (25.6)
***C.jejuni***	*82*	15 (18.3)
***E.histolytica***	*144*	4 (2.8)
**Coinfections**	594	11 (1.8)
**None detected**		410 (78.2)

^a^ Pathogens were not detected in 410 (78.2%) cases.

^b^ Viral pathogens were identified in 154 (29.4%) of stool samples.

**Table 2.** Coinfections of Pathogens

Coinfections	Number
**Adenovirus + Rotavirus**	5
**Adenovirus + Norovirus**	3
**Norovirus + Rotavirus**	1
**Adenovirus + ** ***Salmonella*** ** spp.**	1
**Adenovirus + Rotavirus + ** ***Salmonella*** ** spp.**	1
**Total**	11

**Figure 1.**
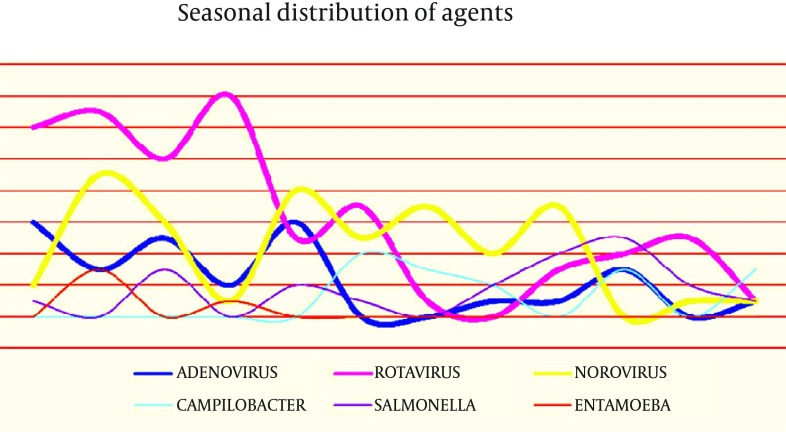
Seasonal Distribution of Acute Gastroenteritis Agents

There was a peak incidence in the first four months of the year for the rotavirus cases (65.3% of cases occurred from January-April) (Table 3 and Figure 1). The peak number of rotavirus cases, which occurred in April, was statistically significant (P < 0.05), while there were low incidences of cases in July and December. Norovirus was seen more frequently in February, May, July and September; it was not detected in October, and was rarely seen in the other months. Norovirus was the most commonly detected infectious agent in August (P < 0.05) (Table 3). Adenovirus was most common in January and May, and not detected in June and July. In contrast with rotavirus and adenovirus; norovirus was found at significant levels in the hottest months of the year (July, August and September) (P < 0.05). Bacterial agents were mostly seen in summer and autumn. In June, July and August *C. jejuni,* and in September, October and November *Salmonella* spp. were found to be the prominent species (Table 3 and Figure 1). 

**Table 3.** Monthly Distribution of Acute Gastroenteritis Pathogens ^a^

Months	Adenovirus, No.	Rotavirus, No.	Norovirus, No.	*C. jejuni*, No.	*Salmonella* spp, No.	*E. histolytica*, No.
**January**	6	12	2	0	1	0
**February**	3	13	9	0	0	3
**March**	5	10	6	0	3	0
**April**	2	14 ^a^	1	0	0	1
**May**	6	5	8	0	2	0
**June**	0	7	5	4	1	0
**July**	0	1	7 ^a^	3	0	0
**August**	1	0	4 ^a^	2	2	0
**September**	1	3	7	0	4	0
**October**	3	4	0	3	5	0
**November**	0	5	1	0	2	0
**December**	1	1	1	3	1	0
**Total**	28	75	51	15	21	4

^a^ P < 0.05, χ2

Age distribution of acute gastroenteritis cases is documented in Table 4. The cumulative age distribution for norovirus-positive cases was 49% (n = 25) for patients younger than 24 months (P < 0.05) and 88.2% (n = 45) in those younger than five years. For rotavirus-positive cases, 28.0% (n = 21) were younger than 24 months, 57.3% (n = 43) were between 25 - 60 months (P < 0.05), and 85.3% (n = 64) were younger than five years. For adenovirus-positive cases, 32.1% (n = 9) were younger than 24 months, and 85.7% (n = 24) were younger than five years. When the dataset was analyzed according to the age groups, rotavirus and norovirus were the leading agents in the first year (n = 55, 30%) (Table 5). For the first 24 months (n = 25, 42.4%), norovirus was the main pathogen (P < 0.05), whereas for ages 25 - 60 months (n = 43, 43%), it was rotavirus (P < 0.05). Furthermore, rotavirus was the main pathogen for children older than five years, but this difference was not significant (P > 0.05). 

In the two-five years age group (n = 100, 54.3%), the cases of viral agents (n = 77, 77%) outnumbered the bacterial agents (n = 22, 22%), and *E. histolytica* was detected in three cases. The majority of the viral agents detected were rotavirus (43%), and the greatest number of bacterial agents was *Salmonella* spp. (63.6%). There were 25 cases over the age of five years, and in 21 (84%) cases, the agents were viruses, with rotavirus (n = 11, 39.3%) being the most common. In this group, the number of bacterial agents was seven, five of which were *Salmonella* spp.

**Table 4.** Age Distribution of Pathogens

Age groups, mo	Number	Pathogens, No. (%)						
		AdV ^a^	RoV ^a^	NoV ^a^	*C. jejuni*	*Salmonella* spp.	*E. histolytica*	Coinfections
**0 - 24**	59	9 (32.1)	21 (28.0)	25 ^b^ (49.0)	5 (33.3)	2 (9.5)	2 (40.0)	5 (45.4)
**25 - 60 **	100	15 (53.6)	43 ^b^ (57.3)	20 (39.2)	8 (53.3)	14 (66.7)	3 (60.0)	3 (27.3)
**> 60**	25	4 (14.3)	11 (14.7)	6 (11.8)	2 (13.4)	5 (23.8)	0	3 (27.3)
**Total**	184	28 (100)	75 (100)	51 (100)	15 (100)	21 (100)	5 (100)	11 (100)

^a^ Abbreviations: AdV, Adenovirus; NoV, Norovirus; RoV, Rotavirus

^b^ P < 0.05, χ2

**Table 5.** Distribution of Age Groups According to Pathogens

Pathogens	Age Groups, No. (%)		
	0 - 24 Months	25 - 60 Months	> 60 Months
**Adenovirus (n = 28)**	9 (15.2)	15 (15)	4 (16)
**Norovirus (n = 51)**	25 (42.4) ^a^	20 (20)	6 (24)
**Rotavirus (n = 75)**	21 (35.6)	43 (43) ^a^	11 (44)
***C. jejuni*** ** (n = 15)**	5 (8.5)	8 (8)	2 (8)
***Salmonella*** ** spp. (n: 21)**	2 (3.4)	14 (14)	5 (20)
***E.histolytica*** ** (n = 5)**	2 (3.4)	3 (3)	0
**Coinfections (n = 11)**	5 (8.5)	3 (3)	3 (12)
**Total**	59	100	25

^a^ P < 0.05, χ2

## 5. Discussion

Diarrheal disorders in childhood account for a large proportion (18%) of childhood deaths, with an estimated 1.5 million deaths per year, making it the second most common cause of child deaths worldwide. The World Health Organization (WHO) and The United Nations Children's Fund (UNICEF) estimate that almost 2.5 billion episodes of diarrhea occur annually in children younger than five years in developing countries, with more than 80% of these cases occurring in Africa and South Asia (46% and 38%, respectively) (1). Acute gastroenteritis is one of the most common infectious diseases and a significant cause of morbidity after upper respiratory cause infections (1). Viral and bacterial enteropathogens are important causes of childhood gastroenteritis in both developed and developing countries (2, 3). 

Bacteria, viruses, parasites and amoebas, have been reported as etiological agents, including; rotavirus, norovirus, adenovirus, astrovirus, *Salmonella* spp., *C. jejuni*, *Shigella* spp., with differences in prevalence depending on demographical, socio-economic, environmental and geographical factors (1, 4, 5). Viruses affect all age groups, and they can cause sporadic or endemic infections,

which are involved in the majority of acute gastroenteritis of undetermined etiology (1). Determination of the etiology is important for treatment and prognosis (1, 6). Viral gastroenteritis can cause severe epidemics and substantial mortality in underdeveloped and developing countries (7). In recent times the development of immunochromatography, enzyme immunoassays, and real time PCR assays for such pathogens, has led to increased sensitivity of viral detection and increasing knowledge of disease epidemiology.

Worldwide, rotaviruses cause the majority of viral acute gastroenteritis in childhood, especially in the first five years of life (7, 8). Overseas studies have reported the incidence of rotavirus gastroenteritis to be between 11% - 77.1%, and 9.9% - 39.8% in national studies (8). Worldwide (7), group A rotavirus are the most frequent viral agent detected in children from Greece and Italy (7-9). Only one study, sourced from Guatemala, showed a threefold difference of enteric adenovirus cases over rotaviruses, which are possibly due to differences in climate (10). 

The incidence of adenovirus gastroenteritis in childhood was reported to be 4.1% by Ahluwalia, 3.6% by Crotti, and 3% by Wilhelmi et al. (5, 11, 12). In Turkey, the incidence was reported as 7.8% by Tunger, 4.7% by Gul, and 16.2% by Bicer et al. (4, 13, 14). As reported in Greece and South Korea, our results confirmed norovirus as the second most common viral agent among hospitalized children with acute gastroenteritis (9.7%) (9, 15). The reported rate of norovirus among stool samples from children with acute sporadic gastroenteritis worldwide ranges between 4.5% and 48.4%, depending on the ages of the children, the setting of the study (outpatients or hospitalized children), and the methodology employed (16). In this study, the reported incidence of rotavirus was 12.6%, and adenovirus 4.71% among 594 children, and norovirus 9.7% among 524 children up to 16 years. Our findings were similar to those of other studies.

Annual seasonality of enteric viruses varies with climate worldwide. A higher prevalence of rotavirus-caused diseases is found in colder temperatures, and a relatively low humidity and dry weather were found in several studies (12, 17, 18). This may be associated with families staying indoors in cold weather, leading to an increase in contact transmission, as the dry conditions encourage aerosol formation of virus-laden particles from patients’ feces. The highest proportion of rotavirus cases were observed in winter and spring (9, 12, 15, 17, 19). In temperate countries, rotavirus is usually seen during the cooler months of fall, winter and spring (12, 20). Seasonal distribution of rotavirus showed a peak incidence in the first four months of the year in our study. The incidence of rotavirus was lowest during the hottest months. Adenoviruses are often seen in summer (51.1%) and spring (9, 15, 21). In our study, adenovirus was most frequently found in January and May, whereas it was not detected in June and July. Norovirus was detected throughout the year, but the rate of isolation was higher in the winter - spring period (9, 15, 20). In contrast with rotavirus and adenovirus, norovirus was the most dominant viral agent in this study during the hottest months of the year (July, August and September).

Bacterial pathogens were reported during all seasons, but salmonellosis and campylobacteriosis were more frequent during spring and summer (12). Our data showed that bacterial agents were seen more often in summer and autumn. *C. jejuni* were the most frequent species in June, July and August, while *Salmonella* spp. were found to be prominent in September, October and November.

Clinically, it may be difficult to differentiate viral gastroenteritis from other forms of the disease; thus, methods to confirm viral etiology such as electron microscopy, culture, antigen detection through enzyme immunoassay, latex agglutination and RT-PCR, are necessary (4, 7, 9, 21, 22). Electron microscopy is a fast and reliable technique, but it is not widely used in practice (22). Enzyme immunoassay (ELISA) and immunochromatography methods are basic and widely used as diagnostic tools for group A rotavirus antigen detection. The sensitivity and specificity of ELISA are 95% and 99%, respectively (22, 23). Recently, the immunochromatography method has gained popularity since its results are readily available in 10 minutes, can be run with a small amount of sample, and it has high sensitivity (93% - 100%) (7, 21, 24). 

The previously mentioned tests may be unreliable in newborns and patients with underlying intestinal problems because of false positivity rates (25). In national clinical studies, this method showed 20.6% - 32% positivity for rotavirus (1, 4, 14, 24, 26), whereas the ELISA showed 39.8% positivity (27). Yaman et al. reported 20.9% positivity for ELISA and 25.65% positivity for latex agglutination (28). Tunger at al. reported 17.4% positivity using an ELISA method (13). Molecular techniques enhanced the overall diagnostic efficacy by 2.5%, and by 10% each for rotavirus and adenovirus (14). In our clinic, immunochromatography resulted in 12.6% of the samples testing positive for rotavirus antigens. This result was consistent with other studies that used the same method (4, 14, 26, 29).

Rotavirus infections are most common in the first two years of life (30). National studies investigating rotavirus infection in the first five years have shown positivity rates of 26.3% - 65.4% during the first year and 46% - 88.9% during the first two years (1, 6). In Taiwan, rotavirus cases are more commonly seen at 0 - 24 months (55%) (20). These reports are inconsistent with our findings. In our study, among the 75 cases with rotavirus infection, 28% of cases were found up to two years and 57.3% were between two and five years. Specific immunizations can decrease the rates of infection during the early ages.

It is generally thought that norovirus is a major pathogen leading to diarrheal outbreaks, causing symptomatic infections in older children and adults. However, some studies have demonstrated that norovirus also has an impact on children younger than two years, which is consistent with the findings of the current study (31, 32). Norovirus infections were mostly seen in our cases aged younger than two years. In other studies, norovirus infections showed positivity rates of 17.3% in Spain and 14.6% in Taiwan during the first five-years-of-age (16, 20). In Taiwan, norovirus was more commonly seen in cases at 0 - 36 months (84.7%) and 0 - 24 months (67.4%) (20). In our study, among the 51 patients with norovirus infections, 49% were up to two years, and 39.2% were between two and five years. Although adenovirus infections can be seen at all ages, similar to rotavirus infections, they are more commonly seen in children under two years (29, 30). In our study, more than half (53.6%) of the enteric adenovirus infections were seen between two and five years. These findings were inconsistent with those reported in the literature.

Bacterial pathogens were reported with various incidences in the literature. Monobacterial infections were detected in 8.4% of the total; *Salmonella* spp. was detected in 258 of the fecal samples in Greece (9). In an Italian study, the reported detection rates of *Salmonella* spp. were 9.9% - 16.4% (8, 12). Among patients in national studies with bacterial gastroenteritis, the detection rate of *Salmonella* spp. and *C. jejuni* were reported to be 2% - 11% and 1% - 13%, respectively (23, 29, 33). Bacterial enteric pathogens of acute gastroenteritis cases diagnosed in this study were *Salmonella* spp. and *C. jejuni*. The detection rates of *Salmonella* spp. and *C. jejuni* were 25.6% (21/82) and 18.3% (15/82), respectively. Bacterial pathogens have been reported at all ages, but salmonellosis (51.8%) and campylobacteriosis (52.6%) were more frequent between one and three years (12). In our study, among the 36 patients with acute bacterial gastroenteritis, 50% were up to four years, and 50% were between four and 16 years.

Coinfections can also be seen, mostly in the form of dual viral infections rather than bacterial-viral coinfections (3, 7, 9, 34-36). Among those, rotavirus and astrovirus as well as rotavirus and adenovirus coinfections were mostly observed, and the rate of rotavirus and adenovirus coinfections were reported to be between 1.2% - 8.2% in various studies (3, 9, 14, 36). In our study, the rates of coinfections (1.9% of the total and 7.6% of the detected agents) were consistent with those reported from other European studies. Our study showed coinfections of rotavirus and adenovirus, rotavirus and norovirus, adenovirus and norovirus, adenovirus and rotavirus, and *Salmonella* and adenovirus/*Salmonella* gastroenteritis.

Viral etiology can be diagnosed with immunochromatography, which is a simple and fast test and this may help to determine the clinical plan and avoid unnecessary antibiotic usage. Molecular techniques (such as RT-PCR) can be used to enhance the overall diagnostic efficacy of viral and bacterial pathogens. Epidemiological studies can also promote vaccination rates for rotavirus, which significantly decrease morbidity and hospitalization rates (37).

It is concluded that, viruses were the major pathogens in all age groups in this study. Rotavirus was the most frequent agent of acute gastroenteritis in Turkish children for ages over two years. Norovirus was the second most common etiological agent, which mainly affects children less than two-years-of-age. The incidence of rotavirus was high during winter and early spring, whereas during late spring, summer and early autumn, norovirus was more frequently notified. In January, February, March, April, June, October and November, rotavirus was the dominant agent; whereas in May, July, August and September, norovirus was reported. Adenovirus, being the other cause of viral acute diarrhea, was not stated as the leading pathogen at any time of the year, although all three agents had the same incidence in December. Bacterial agents dominated viral pathogens only in October and December. In October, *Salmonella* spp. and in December, *C. jejuni* were the most common agents causing acute gastroenteritis.
